# The Treatment of Whooping Cough

**Published:** 1887-01

**Authors:** 


The Treatment of Whooping-Cough.—In his summary of treatment, in a clinical lecture delivered at the Philadelphia Hospital (*Medical News*), Dr. John M. Keating emphazies the value of the steam spray and atomization of medicated solutions, among which he ascribes value to Dobell's solution, eucalyptol, and thymol. With the bichloride he advises caution. Corrosive sublimate, which is now used for almost everything, he says, has also been applied here in the form of the spray. He remarks that it is a dangerous drug to put into the hands of an inexperienced person, and, as we have so many other useful remedies for this affection, he thinks it wise to avoid the use of corrosive sublimate. He has used listerine extensively with good results in the treatment of whooping-cough. He employs it in the strength of one drachm to two ounces of water, with an ordinary hand atomizer, directs the nurse to apply it twelve or more times a day, and finds that little children, even babies, do not object to it. He adds to it tincture of belladonna, potassium carbonate, or ammonium bromide, as the case may demand. Chloride of ammonium he also finds of great service in the form of spray.—*New York Medical Journal.*

